# Supplementary material for: Chemical composition and lipid profile of mare colostrum and milk of the quarter horse breed
Source: PLoS One. 2020 Sep 14;15(9):e0238921. doi: 10.1371/journal.pone.0238921 (PMC7489553; doi:10.1371/journal.pone.0238921)
Supplement: Attachment — Submitted filename: PONE-D-20-04990_reviewer.pdf [file pone.0238921.s001.pdf]

PLOS ONE

Chemical composition and lipid profile of mare colostrum and milk of the quarter horse breed

--Manuscript Draft--

**Manuscript Number:**	PONE-D-20-04990
**Article Type:**	Research Article
**Full Title:**	Chemical composition and lipid profile of mare colostrum and milk of the quarter horse breed
**Short Title:**	composition of mare colostrum and milk of the quarter horse breed
**Corresponding Author:**	Bruna M E Chagas<br>Universidade Federal do Rio Grande do Norte Centro de Ensino Superior do Serido<br>Natal, BRAZIL
**Keywords:**	Unsaturated fatty acids, lactation stage, equidae.
**Abstract:**	The objective of this study was to characterize the chemical composition and lipid profile of colostrum and milk of purebred Quarter Horse mares. Thirty-four (34) purebred mares were selected, which were then separated into groups according to age, birth order and lactation stage. Colostrum samples were collected in the first six hours after delivery and milk samples from the 7 th postpartum day, with intervals of 14 days until the end of lactation. The samples were refrigerated and sent to the Milk Laboratory of the University (Laboleite - UFRN), where they were analyzed for chemical composition. Colostrum was assessed by refractometry. The lipid profile was determined by gas chromatography through a separation of methyl esters. The data were tabulated and submitted to descriptive statistics and analysis of variance by the F-Test, and the groups were compared by the Tukey test using a significance level of 5%. There was a high protein content and reduced lactose content for the colostrum of the Quarter Horse mares. The milk composition was not influenced by the mares' age. However, variations in the lactation stage and in the birth order of the Quarter Horse mares altered the milk's chemical composition. There is variation in the lipid composition of milk according to the lactation stage, without changing the characteristic profile of the mares' milk or diminishing the nutritional quality of the lipid fraction.
**Order of Authors:**	Ícaro M. L. G. Barreto
	Stela A. Urbano
	Chiara A. A. Oliveira
	Cláudia S. Macêdo
	Luiz H. F. Borba
	Bruna M E Chagas
	Adriano H. N. Rangel
**Additional Information:**	
**Question**	**Response**
**Financial Disclosure**<br><br>Enter a financial disclosure statement that describes the sources of funding for the work included in this submission. Review the submission guidelines for detailed requirements. View published research articles from *PLOS ONE* for specific examples.	The author(s) received no specific funding for this work.

Powered by Editorial Manager® and ProduXion Manager® from Aries Systems Corporation

This statement is required for submission and **will appear in the published article** if the submission is accepted. Please make sure it is accurate.<br><br>**Unfunded studies**<br>Enter: *The author(s) received no specific funding for this work.*<br><br>**Funded studies**<br>Enter a statement with the following details:<br>• Initials of the authors who received each award<br>• Grant numbers awarded to each author<br>• The full name of each funder<br>• URL of each funder website<br>• Did the sponsors or funders play any role in the study design, data collection and analysis, decision to publish, or preparation of the manuscript?<br>• **NO** - Include this sentence at the end of your statement: *The funders had no role in study design, data collection and analysis, decision to publish, or preparation of the manuscript.*<br>• **YES** - Specify the role(s) played.<br><br>* typeset	
**Competing Interests**<br><br>Use the instructions below to enter a competing interest statement for this submission. On behalf of all authors, disclose any competing interests that could be perceived to bias this work—acknowledging all financial support and any other relevant financial or non-financial competing interests.<br><br>This statement **will appear in the published article** if the submission is accepted. Please make sure it is accurate. View published research articles from *PLOS ONE* for specific examples.	The authors have declared that no competing interests exist.

**NO authors have competing interests**<br><br>Enter: *The authors have declared that no competing interests exist.*<br><br>**Authors with competing interests**<br><br>Enter competing interest details beginning with this statement:<br><br>*I have read the journal's policy and the authors of this manuscript have the following competing interests: [insert competing interests here]*<br><br>* typeset	
**Ethics Statement**<br><br>Enter an ethics statement for this submission. This statement is required if the study involved:<br><br>• Human participants<br>• Human specimens or tissue<br>• Vertebrate animals or cephalopods<br>• Vertebrate embryos or tissues<br>• Field research<br><br>Write "N/A" if the submission does not require an ethics statement.<br><br>General guidance is provided below. Consult the submission guidelines for detailed instructions. **Make sure that all information entered here is included in the Methods section of the manuscript.**	The authors have declared that Ethics Statement is not required.

**Format for specific study types**<br><br>**Human Subject Research (involving human participants and/or tissue)**<br>• Give the name of the institutional review board or ethics committee that approved the study<br>• Include the approval number and/or a statement indicating approval of this research<br>• Indicate the form of consent obtained (written/oral) or the reason that consent was not obtained (e.g. the data were analyzed anonymously)<br><br>**Animal Research (involving vertebrate animals, embryos or tissues)**<br>• Provide the name of the Institutional Animal Care and Use Committee (IACUC) or other relevant ethics board that reviewed the study protocol, and indicate whether they approved this research or granted a formal waiver of ethical approval<br>• Include an approval number if one was obtained<br>• If the study involved *non-human primates*, add *additional details* about animal welfare and steps taken to ameliorate suffering<br>• If anesthesia, euthanasia, or any kind of animal sacrifice is part of the study, include briefly which substances and/or methods were applied<br><br>**Field Research**<br><br>Include the following details if this study involves the collection of plant, animal, or other materials from a natural setting:<br>• Field permit number<br>• Name of the institution or relevant body that granted permission	
**Data Availability**<br><br>Authors are required to make all data underlying the findings described fully available, without restriction, and from the time of publication. PLOS allows rare exceptions to address legal and ethical concerns. See the PLOS Data Policy and FAQ for detailed information.	Yes - all data are fully available without restriction

A Data Availability Statement describing where the data can be found is required at submission. Your answers to this question constitute the Data Availability Statement and **will be published in the article**, if accepted.<br><br>**Important:** Stating 'data available on request from the author' is not sufficient. If your data are only available upon request, select 'No' for the first question and explain your exceptional situation in the text box.<br><br>Do the authors confirm that all data underlying the findings described in their manuscript are fully available without restriction?	
**Describe where the data may be found in full sentences. If you are copying our sample text, replace any instances of XXX with the appropriate details.**<br><br>• If the data are **held or will be held in a public repository**, include URLs, accession numbers or DOIs. If this information will only be available after acceptance, indicate this by ticking the box below. For example: *All XXX files are available from the XXX database (accession number(s) XXX, XXX.).*<br>• If the data are all contained **within the manuscript and/or Supporting Information files**, enter the following: *All relevant data are within the manuscript and its Supporting Information files.*<br>• If neither of these applies but you are able to provide **details of access elsewhere**, with or without limitations, please do so. For example:<br><br>*Data cannot be shared publicly because of [XXX]. Data are available from the XXX Institutional Data Access / Ethics Committee (contact via XXX) for researchers who meet the criteria for access to confidential data.*<br><br>*The data underlying the results presented in the study are available from (include the name of the third party*	All relevant data are within the manuscript and its Supporting Information files.

and contact information or URL).<br>• This text is appropriate if the data are owned by a third party and authors do not have permission to share the data.<br><br>* typeset	
Additional data availability information:	Tick here if the URLs/accession numbers/DOIs will be available only after acceptance of the manuscript for publication so that we can ensure their inclusion before publication.

Powered by Editorial Manager® and ProduXion Manager® from Aries Systems Corporation

Manuscript Click here to download Manuscript renamed_e9be8.docx

Chemical composition and lipid profile of mare colostrum and milk of the quarter horse breed

Ícaro M. L. G. Barreto[1¶], Stela A. Urbano[1¶], Chiara A. A. Oliveira[2¶], Cláudia S. Macêdo[1&], Luiz H. F. Borba[1&], Bruna M E Chags[2&], Adriano H. N. Rangel[1¶]

[1]Agricultural School of Jundiaí, Federal University of Rio Grande do Norte, Natal, RN, Brazil.
[2]School of Veterinary Medicine and Zootechnics - Federal University of Bahia, Salvador, BA, Brazil
[3]Department of Systematic Ecology, Infrastructure Superintendence, Federal University of Rio Grande do Norte, Natal, RN, Brazil.

*Corresponding author
E-mail: brunam.emerenciano@gmail.com (BMEC)

[¶] These authors contributed equally to this work.
[&] These authors also contributed equally to this work.

Abstract

The objective of this study was to characterize the[illegible]mical composition and lipid profile of colostrum and milk of purebred Quarter Horse mares. Thirty-four (34) purebred mares were selected, which were then separated into groups according to age, birth order and lactation stage. Colostrum samples were collec[illegible] the first six hours after delivery and milk samples from the 7th postpartum day, with intervals of 14 days until the end of lactation. The samples were refrigerated and sent to the Milk Laboratory of the University (*Laboleite - UFRN*), where they were analyzed for chemical composition. Colostrum was assessed by refractometry. The lipid profile was determined by gas chromatography through a separation of methyl esters. ~~The data were tabulated and submitted to descriptive statistics and analysis of variance by the F-Test, and the groups were compared by the Tukey test using a significance level of 5%.~~ There was a high protein content and reduced lactose content for the colostrum of the Quarter Horse mares. The milk composition was not influenced by the mares' age. However, variations in the lactation stage and in the birth order of the Quarter Horse mares altered the milk's chemical composition. There is variation in the lipid composition of milk according to the lactation stage, without changing the characteristic profile of the mares' milk or diminishing the nutritional quality of the lipid fraction.

Keywords: Unsaturated fatty acids, lactation sta[illegible]quidae.

Introduction

A considerable number of horses have been bred in several countries around the world in order to produce milk [1] because of its nutritional and therapeutic properties. Mares' milk is consumed by 30 million people worldwide, and has been studied as a milk substitute in newborns and pre-mature humans [2], in addition to being frequently used as a dietary supplement for older adults, recovering patients, and mainly children allergic to cow milk [1].

On average, equine milk is composed of 6.5% lactose, 1.8% protein, 1.0% fat and 440 kcal/kg of energy [3]. It presents a desired protein profile in human food due to the whey:casein protein ratio and the spongiform structure of the micelles, which make it physiologically more digestible than cow milk [4]. The nutritional quality of the lipid fraction of equine milk is composed of small amounts of stearic and palmitic acids and high amounts of linoleic and linolenic acids [5], which has also supported indications for supplying equine milk to humans.

Age, birth order, body weight of the mares, diet, environmental conditions and lactation stage have an influence on the chemical composition of milk [5, 6]. In addition to these factors, race and genetics can change the composition of equine milk, especially the protein, fat and lactose levels [7]. Thus, the objective of this study was to characterize the chemical composition and lipid profile of colostrum and milk from purebred Quarter Horse mares of different ages, birth orders and lactation stages.

Materials and methods

Animal ethics and experimentation

The trial was submitted to evaluation by the Ethics Committee on the Use of Animals at *UFRN* (protocol 062/2017), receiving approval registered under opinion number 058.062/2017. All animal management practices followed the recommendations of the National Council for the Control of Animal Experimentation (*CONCEA*) for the protection of animals used for animal experimentation and other scientific purposes, in accordance with the provisions of Law No. 11,794, of 8 October 2008, of Decree No. 6,899, of July 15, 2009.

Experimental animals, material sampling and laboratory analysis

Thirty-four (34) purebred mares were selected from three different stud farms specialized in breeding and selection of Quarter Horses in Rio Grande do Norte state. The mares were separated into groups according to age, birth order and lactation stage. The collections took place between the months of July/2017 and September/2018.

Colostrum samples were collected after delivery, not exceeding six hours after the event, and were obtained by manual milking after cleaning the udder and stored in previously sterilized plastic bottles. The composition (fat, protein, casein, lactose, total solids and defatted dry extract), refractometry (Brix percentage determination) and lipid profile of the colostrum samples were analyzed.

Milk samples were collected from the 7th postpartum day with 14-day intervals, and thus continued until the end of lactation (180 days after delivery). The foal remained separated from the mare during the last two hours preceding the procedure on the day determined for collection in order to guarantee there being a sufficient volume of milk for sampling accumulated in the udder. The mares' udders were previously sanitized with a compress soaked with 70% alcohol, while the milker's hands were washed with clean water and neutral soap, dried with paper

towels and also sanitized with 70% alcohol. The first three jets of milk were discarded at the time of collection, and then the udder was fully milked, with the milk being placed in a glass container previously sterilized in an autoclave.

The samples were identified, placed in an isothermal box containing artificial ice (4 to 8ºC) and sent to the *UFRN* Milk Quality Laboratory (*LABOLEITE*). The samples were subjected to electronic analysis by infrared absorption in DairySpec FT Bentley equipment to determine the chemical composition of the milk and colostrum. Qualitative analysis of colostrum was performed using a portable optical refractometer for sugar (Kasvi®, model K52-032, with a measurement range of 0 to 32% Brix and minimum division of 0.2%) after calibrating it with distilled water, as recommended by the manufacturer. One drop of colostrum was placed on the refractometer prism with the sample at room temperature and homogenized, and then reading was conducted through the monocular lens. The result in Brix% was obtained by the separation between the light area and the dark area formed on the equipment display after perpendicular disposition of the equipment to light.

The milk samples were lyophilized and the fatty acid methyl esters were obtained by adapting the methodology proposed by Kramer [8] to analyze the lipid profile. Approximately 0.8g of samples were weighed in glass tubes (16 x 150mm) with screw caps and septums in order to contain 15 to 30 mg of fat. Next, 2mL of hexane and 2mL of sodium methoxide (0.5M in methanol) were added to the tubes, followed by vortexing (30 seconds) and heating in a water bath (50°C for 10 minutes). The tubes were subsequently cooled in running water and 3 ml of acetyl chloride (5% in methanol) were added, which was again heated (80°C for 10 minutes). Then, 1 ml of hexane and 10 ml of 6% K_2CO_3 were added, followed by vortexing for 1 minute and centrifugation (4,000 rpm for 2 minutes). The supernatant was transferred to 15mL Falcon tubes with approximately 1g of Na_2SO_4 mixture (previously oven dried) and activated carbon

(1:1), followed by stirring (1 min.) and centrifugation (1 min. 4000 rpm). The supernatant was collected, transferred to an amber vial and then stored in a freezer at -20ºC.

The separation of the methyl esters from fatty acids was performed in a gas chromatograph (Focus GC - Thermo Scientific) equipped with flame ionization detector (CG-DIC) and SPTM-2560 capillary column (100m x 0.25mm x 0.20 µm - Supelco). The analysis parameters were: injector temperature of 250°C; detector temperature of 280°C; and 30:1 split ratio. The oven temperature was initially set at 140°C, increasing at a heating rate of 1°C/min to 220°C; then remaining at that temperature for 25 minutes. Hydrogen gas was used as carrier gas at a flow rate of 1.5 mL/minute. The injections were performed in duplicates for each extraction and the injection volume was 1µL. The identification of the fatty acid methyl esters was performed by comparing the peak retention times of the samples with the retention time of the esters of the reference standard (GLC-674, Nu-Chek Prep, Inc.), and the result was obtained through normalizing the areas with the results expressed as a percentage.

The atherogenicity index (AI) and thrombogenicity index (TI) were calculated using the equation described by Ulbricht and Southgate (1991):

$$AI = (C12{:}0 + 4 \times C14{:}0 + C16{:}0) / [\Sigma MUFA + \Sigma(n\text{-}6) + \Sigma(n\text{-}3)]$$

$$TI = (C14{:}0+C16{:}0+C18{:}0) / [0.5\times\Sigma MUFA+0.5 \times \Sigma(n\text{-}6) +3 \times \Sigma(n\text{-}3) + \Sigma(n\text{-}3) / \Sigma (n\text{-}6)]$$

Statistical procedures

Data were tabulated in spreadsheets and submitted to descriptive statistics and analysis of variance by the F-test. The groups were divided according to age, birth order and lactation stage, and were then compared using the Tukey test at a significance level of 5% for type I error. Only the different lactation stages were compared for the lipid profile analysis of milk.

The statistical analyzes were performed using the SAS (Statistical Analysis System) statistical package, and the analysis of variance was performed according to the following model:

$$Y_{ij} = \mu_i + group_j + residual_{ij}$$

In which:

Y_{ij} = dependent variables;

μ_i = overall mean;

$group_j$ = effect of the jth group (age, birth order and lactation stage) on dependent variables, being group 1 to 3;

$residual_{ij}$ = residual effect.

Results and discussion

The values verified for the colostrum composition (Table 1) confirm its nutritional richness, in which it is important to highlight the high percentage of protein found for the colostrum of Quarter Horse mares (18.06%) was higher than the average of 15% reported by Csapó et al. [9] for Hungarian Draught, Haflinger, Breton and Boulonnaise mares; and the 16% found by Pecka et al. [10] when they evaluated the colostrum of Arabian mares. The lactose content of the colostrum evaluated in this study also differed from those presented by other authors, such as: 3.4% cited by Salimei et al. [11]; 2.95% found by Pikul & Wójtowski [12]; and 2.46% presented by Pecka et al. [10]. The results suggest that the colostrum of Quarter Horse mares may contain more protein and be less dense in energy when compared to other breeds.

The °Brix values obtained were high, following the high protein content of the evaluated material, since approximately 80% of the colostrum protein corresponds to immunoglobulins

[9]. The analyzed colostrum samples fall within the range of 20 to 30% of the refractive index established by Nath et al. [13], which classifies them as good, and constitutes an aspect of relevant importance for passive transfer of immunity and consequently for establishing the newborn's health.

Table 1. Chemical composition of colostrum and milk from purebred Quarter Horse mares.

Variable (%)	Colostrom		Milk	
	Mean ± SD	CV (%)	Mean ± SD	CV (%)
Fat	1.70 ± 1.05	61.31	0.73 ± 0.45	61.30
Total protein	18.06 ± 2.00	11.09	1.68 ± 0.26	15.44
Casein	13.66 ± 2.00	14.63	1.26 ± 0.20	16.20
Lactose	1.53 ± 0.53	34.81	6.62 ± 0.30	4.45
Total solids	20.49 ± 2.36	11.53	10.00 ± 0.59	5.90
DDE[1]	19.95 ± 1.72	8.66	9.30 ± 0.27	2.89
Brix%	27.40 ± 4.15	15.18	-	-

[1]Defatted dry extract; CV = coefficient of variation; SD: standard deviation.

When analyzing the chemical characterization of milk (Table 1), a reversal between the protein and lactose concentrations is noticeable when the two milk secretions (colostrum and milk) are compared. However, as lactose does not have as high concentrations in milk as protein does in colostrum, the levels of total solids and defatted dry milk extract are considerably lower than those observed for colostrum.

In studies conducted with Quarter Horse mares, Gibbs et al. [14] and Burns et al. [15] reported a variation of 1.8 to 2.9% for total milk protein, constituting values close to those found in this study. Lactose is a component which naturally stands out in the composition of equine milk [16], and the values verified remained in the average reported by the literature (6.1 to 7.3%) for the most diverse breeds studied, as reported by Santos et al. [17], Reis et

al.[18], Pecka et al. [10], Markiewicz-Keszycka et al. [19], showing the preponderance of lactose as a source of carbohydrate in m[illegible]’ milk [10].

The value regarding fat content found in this study is less than the range of 1.0-1.5% reported by Gibbs et al. [14] for Quarter Horse mares, and also below the average value of 1.25% reported by Salamon et al. [5], but are higher than the 0.62% reported by Reis et al. [18] for milk from Mangalarga mares. Equine milk certainly has low levels of fat when compared to milk from other species [2]; however, the measurement of this component in mares’ milk is surrounded by methodological details which are difficult to control and which translate into the high coefficient of variation (61.30%) presented in Table 1, and therefore deserve a brief discussion.

The small cistern of the mare’s udder requires frequent milking and/or breastfeeding throughout the day, more specifically every 2 or 3 hours [20], and in addition to this particularity, an accumulation of milk in the breast socket, which requires a reasonable release of oxytocin for ejection [21]. When extrapolated to the sample collection methodology, these anatomical and physiological particularities reflect on the difficulty of completely emptying the udder, which is directly related to the fat content of milk [22], since the residual fraction milk is rich in fat. Therefore, it is possible that the low fat content of milk found in this study is not solely and exclusively explained by genetic variations, but also because there is not enough oxytocin release during the sample collection to remove the residual milk fraction, resulting in in low-fat samples.

There was a significant effect of the lactation stage on the fat, total protein and casein levels, with the effect of such variations also occurring on the defatted dry extract levels (Table 2).

Table 2. Effect of the lactation stage on the milk composition of purebred Quarter Horse mares.

Variable (%)	Days in lactation		
	7 - 60	61 - 120	121 - 180
Fat	0.61 ± 0.41^{b}	0.85 ± 0.54^{a}	0.70 ± 0.36^{ab}
Total protein	1.94 ± 0.31^{a}	1.69 ± 0.18^{b}	1.53 ± 0.18^{c}
Casein	1.47 ± 0.24^{a}	1.27 ± 0.14^{b}	1.15 ± 0.15^{c}
Lactose	6.62 ± 0.23	6.60 ± 0.30	6.64 ± 0.32
Total solids	10.11 ± 0.50	10.09 ± 0.73	9.86 ± 0.46
Defatted dry extract	9.49 ± 0.26^{a}	9.29 ± 0.25^{b}	9.20 ± 0.24^{b}

$^{a, b, c}$ Different letters on the same line indicate statistical difference by the Tukey test ($p<0.05$).

According to Markiewicz-Keszycka et al. [19], the evolution of lactation in mares leads to producing a milk which is rich in lactose, but low in fat, protein and total solids. The fat levels at the end of lactation in this study were higher than those found at the beginning; however, they were lower than the 0.9% reported by Burns et al. [15] for milk from Quarter Horse mares at 150 days of lactation, thus reaffirming the difficulty of completely emptying the udder during collection and the permanence of alveolar milk in the mares evaluated in this study. Regarding protein contents and their fractions, there was a gradual reduction during lactation exactly as reported by Salimei and Fantuz [22]: a decrease of 20 to 25% of the total protein between the 28th and 150th days of lactation, accompanied by a 20 to 30% decrease of casein within the same period.

It is noteworthy that variations in the milk composition throughout lactation are essential for adjustments in the nutritional management of foals given the early development and rapid growth of horses at this stage of life (NRC, 1989). Add to this physiological nature,

the expressive and particular muscular development of the Quarter Horse breed, which certainly requires an increase in protein intake so that there is no nutritional deficit or consequently losses in animal performance.

There was a difference ($p<0.05$) for the lactose levels in the effects of the birth order on the milk composition (Table 3), with a notable decrease in this component according to the maturity of the glandular breast tissue. Similar behavior was observed for defatted dry extract, most likely as a result of lactose variation, since this component is part of the dry extract.

Table 3. Effect of birth order on the milk composition of purebred Quarter Horse mares.

**Variable (%)**	**Birth order**					
	**1**	**2**	**3**	**4**	**5**	**6**
Fat	0.75 ± 0.52	0.73 ± 0.40	0.61 ± 0.42	0.84 ± 0.52	0.76 ± 0.39	0.76 ± 0.46
Prot	1.62 ± 0.14	1.62 ± 0.18	1.74 ± 0.33	1.61 ± 0.22	1.67 ± 0.23	1.74 ± 0.30
Cas	1.23 ± 0.12	1.22 ± 0.15	1.31 ± 0.24	1.21 ± 0.18	1.26 ± 0.19	1.30 ± 0.24
Lact	6.82 ± 0.24^{a}	6.70 ± 0.30^{ab}	6.67 ± 0.29 ^{abc}	6.48 ± 0.27^{c}	6.52 ± 0.26^{bc}	6.59 ± 0.29^{bc}
TS	9.98 ± 0.89	10.09 ± 0.42	9.86 ± 0.68	10.06 ± 0.62	10.01 ± 0.43	10.02 ± 0.45
DDE	9.38 ± 0.16^{a}	9.36 ± 0.20^{ab}	9.34 ± 0.30^{ab}	9.19 ± 0.05^{ab}	9.26 ± 0.22^{ab}	9.27 ± 0.29^{b}

$^{a, b, c}$ Different letters on the same line indicate statistical difference by the Tukey test ($p<0.05$).
Fat = fat; Prot = total protein; Cas = casein; Lact = lactose; TS = total solids; DDE = defatted dry extract.

In evaluating the milk production and composition of primiparous and multiparous Quarter Horse mares, Pool-Anderson et al. [23] found greater production in multiparous mares, but did not report any variation in the milk secretion composition, differently from what occurred in this study. The fact that the lactose content is linked to the osmotic function and the milk production of the mammary gland [24] generated contrary expectations to the observed result, since young mares have lower production, which would lead to smaller lactose content in the milk of low birth order mares.

Although low birth order mares are generally younger animals, there was no effect ($p>0.05$) of age on milk composition (Table 4).

Table 4. Effect of age on the milk composition of purebred Quarter Horse mares.

**Variable (%)**	**Age (years)**		
	**3 - 5**	**6 - 10**	**11 - 19**
Fat	0.77 ± 0.43	0.71 ± 0.47	0.76 ± 0.41
Total protein	1.63 ± 0.11	1.67 ± 0.27	1.71 ± 0.31
Casein	1.23 ± 0.08	1.26 ± 0.21	1.29 ± 0.25
Lactose	6.64 ± 0.29	6.59 ± 0.30	6.66 ± 0.29
Total solids	9.99 ± 0.72	9.97 ± 0.61	10.04 ± 0.45
Defatted dry extract	9.32 ± 0.20	9.29 ± 0.29	9.29 ± 0.26

Milk production in mammals increases with age until physiological maturity is reached [25], when there is a tendency to a functional reduction of the mammary gland caused by aging of the glandular tissue [26]. In addition to these cirumstances is the dilution effect, in which the highest production tends to dilute the dry extract components [24], and so variation in the milk composition from mares of different ages was expected, especially between the evaluated extremes, but this fact did not occur in this study.

Tables 5 and 6 show the lipid composition of colostrum and milk from mares and the effect of the lactation stage on the lipid profile of milk, while Table 8 shows the relationships between fatty acids.

Saturated fatty acids prevailed over unsaturated faty acids in colostrum, with an emphasis on palmitic (C16:0), capric (C10:0), lauric (C12:0) and myristic (C14:0) acids, which increased the sum of saturated fatty acids (Table 5) and consequently the AGS:AGI ratio (Table 7). In the case of unsaturated acids, C18:1n9cis, C18:2n6cis and C18:3n3 stood out in

comparison to the others. Similar behaviors for such acids have been reported by Pikul et al. [12] and Salamon et al. [5] for mares' colostrum; however, the values obtained in this study were lower than those reported by these authors.

Table 5. Means and standard deviations of saturated fatty acids (peak area %) in Quarter Horse mares' colostrum and milk.

Fatty acid	Colostrom	Milk	Milk - days in milk		
			7 – 60 d	61 – 120 d	121 – 180 d
C4:0	0.035±0.019	0.110±0.020	0.122±0.032^{a}	0.110±0.008^{a}	0.097±0.005^{a}
C6:0	0.142±0.052	0.244±0.055	0.292±0.045^{a}	0.255±0.017^{ab}	0.185±0.288^{bc}
C8:0	2.375±0.033	2.514±0.367	2.715±0.028^{a}	2.747±0.101^{a}	2.080±0.167^{b}
C10:0	10.098±0.066	5.674±1.260	6.192±0.972^{a}	6.685±0.045^{a}	4.145±0.191^{b}
C12:0	8.742±0.083	6.098±0.856	5.882±0.730^{b}	7.075±0.025^{a}	5.335±0.206^{b}
C14:0	6.612±0.243	6.560±0.639	6.095±0.561^{b}	7.312±0.123^{a}	6.272±0.109^{b}
C16:0	19.16±0.472	21.401±1.227	20.295±0.954^{ab}	22.437±0.162^{a}	21.485±1.229^{ab}
C18:0	4.282±0.548	1.626±0.362	1.867±0.286^{a}	1.827±0.080^{a}	1.185±0.051^{b}
∑AGS	53.652±1.364	46.491±3.818	45.322±3.632^{b}	50.795±0.522^{a}	43.357±0.628^{b}

^{a,b,c} Means followed by different letters on the same line differ statistically from each other using the Tukey test at the 5% significance level. ∑AGS = sum of saturated fatty acids.

There was a higher prevalence of unsaturated fatty acids regarding the lipid profile of mature milk when compared to colostrum; however, fatty acids (saturated and unsaturated) which stood out in the lipid profile of colostrum also did so in milk, with the values obtained herein within the threshold presented by Claeys et al. [27]. According to these authors, equine milk in fact has higher proportions of unsaturated fatty acids when compared to milk from other species (especially cattle), due to the minimal occurrence of biohydrogenation before the absorption of unsaturated fatty acids.

There was an effect of the lactation stage on the lipid profile of mares' milk, with higher values of saturated fatty acids being obtained in the middle third of lactation. This is similar to the result presented by Orlandi et al. [28], especially in relation to C12:0, C14:0 and C16:0 acids. Pikul et al. [12] also found that saturated acids decrease with the lactation progress in evaluating the lipid profile of mares' milk throughout lactation. There was a significant reduction in linoleic acid in the final third of lactation for the most important unsaturated acids in our study, while linolenic acid had its values depressed in the middle of lactation. Consequently, the sum of polyunsaturated fatty acids was also lower for the middle third of lactation.

Tabela 6. Means and standard deviations of unsaturated fatty acids (peak area %) in Quarter Horse mares' colostrum and milk.

Fatty acid	Colostrom	Milk	Milk - days in milk		
			7 – 60 d	61 – 120 d	121 – 180 d
C14:1	0.180±0.020	0.554±0.209	0.352±0.106^{b}	0.497±0.005^{b}	0.812±0.037^{a}
C16:1	1.942±0.140	5.494±1.662	4.200±1.172^{b}	4.882±0.056^{b}	7.400±1.085^{a}
C18:1n9cis	15.700±0.186	16.688±0.971	16.64±1.405^{a}	16.600±0.367^{a}	16.822±1.144^{a}
C18:2n6cis	14.517±0.924	14.011±3.655	16.00±2.547^{a}	16.585±0.416^{a}	9.442±0.600^{b}
C20:1n9	0.470±0.014	0.260±0.029	0.282±0.017^{a}	0.245±0.005^{a}	0.252±0.043^{a}
C18:3n3	4.560±0.155	11.415±5.314	12.212±3.44^{a}	5.387±0.075^{b}	16.645±2.442^{a}
C20:2n6	0.520±0.012	0.278±0.043	0.302±0.005^{a}	0.322±0.009^{a}	0.220±0.008^{b}
C20:3n3	0.147±0.015	0.264±0.097	0.275±0.052^{b}	0.150±0.000^{c}	0.367±0.020^{a}
∑AGM	18.697±0.295	23.099±2.547	21.547±2.636^{a}	22.310±0.390^{a}	25.440±2.312^{a}
∑AGPI	19.980±1.041	26.105±3.236	28.985±0.985^{a}	22.560±0.443^{b}	26.770±2.971^{a}

^{a,b} Means followed by different letters on the same line differ statistically from each other using the Tukey test at the 5% level of significance. ∑AGM = sum of monounsaturated fatty acids; ∑AGP = sum of polyunsaturated fatty acids.

The relationships between fatty acids presented in Table 7 show that even though some values obtained in this study are lower than those contained in the literature, there is a relevant

nutritional advantage of the Quarter Horse mares' milk compared to milk of other species. The high linoleic and linolenic acid concentrations, which have important biological functions [2] bring the relationships between fatty acids close to the ideal value.

Table 7. Means and standard deviations of the relationships between saturated and unsaturated fatty acids in colostrum and milk of Quarter Horse mares.

Fatty acid ratios	Colostrom	Milk	Milk - days in milk		
			7 – 60 d	61 – 120 d	121 – 180 d
∑n6	15.177±0.938	14.351±3.697	16.390±2.538[a]	16.947±0.410[a]	9.717±0.605[b]
∑n3	4.707±0.167	11.668±5.382	12.505±3.489[a]	5.537±0.075[b]	16.962±2.373[a]
n6:n3	3.225±0.155	1.692±1.122	1.437±0.603[b]	3.062±0.065[a]	0.577±0.044[b]
AGS:AGI	1.390±0.080	0.954±0.153	0.902±0.136[b]	1.130±0.0316[a]	0.830±0.023[b]
AI	1.415±0.085	1.105±0.173	1.010±0.150[b]	1.312±0.034[a]	0.995±0.040[b]
TI	0.962±0.069	0.597±0.215	0.512±0.129[b]	0.865±0.019[a]	0.415±0.058[b]

[a,b] Means followed by different letters on the same line differ statistically from each other using the Tukey test at the 5% level of significance. ∑n6 = sum of omega 6 fatty acids; ∑n3 = sum of omega 3 fatty acids; n6:n3 = ratio between omega 3 and omega 6 fatty acids; AGS:AGI = ratio between total saturated and unsaturated fatty acids; AI = atherogenicity index; TI = thrombogenicity index.

Lower atherogenicity and thrombogenicity indices indicate the potential for atheroma and thrombus prevention [29] and were very close to those presented by Pikul et al. [30] for Konik mares. The reduction in indices in the final third of lactation was also observed by Markiewicz-Kęszycka et al. [31].

Conclusions

Quarter Horse mares produced colostrum with higher protein content and lower lactose content when compared to other breeds. The milk does not have a composition influenced by the mares' age; however, the lactation stage and the birth order alter the chemical composition of the milk of Quarter Horse mares. There is variation in the milk's lipid composition according

to the lactation stage without changing the characteristic profile of mares' milk and without harming the nutritional quality of the lipid fraction.

Acknowledgements

The authors gratefully acknowledge the Laboratório de Qualidade do Leite from the Universidade Federal do Rio Grande do Norte for chemical analysis and the Haras Bom Pasto in Serrinha City, State of Rio Grande do Norte, Northeast of Brazil for making horse available.

References

1. Bruckmaier RM. Normal and disturbed milk ejection in dairy cows. Domest Anim Endocrinol. 2005; 29: 268–73.

2. Burns HD, Gibbs PG, Potter, GD. Milk-energy production by lactating mares. J Equine Vet Sci. 1992; 12:118-120.

3. Claeys WL, Verraes C, Cardoen S, De Block J, Huyghebaert A, Raes K, Dewettinck K, Herman L. Consumption of raw or heated milk from different species: An evaluation of the nutritional and potential health benefits. Food Control. 2014; 42:188 – 201.

4. Csapó J, Salamon S, Lóki K, Csapó-kiss Z. Composition of mare's colostrum and milk II. Protein content, amino acid composition and contents of macro-and micro-elements. Acta Univ Sapient Ser Aliment. 2009; 2:133-148.

5. Danków R, Wójtowski J, Pikul J, Niznikowski R, Cais-sokolínska D. Effect of lactation on the hygiene quality and some milk physicochemical traits of the Wielkopolska mares. Arch Tierz. 2006; 49:201-206.

6. Doreau M, Martin-Rosset W. Horse. Encyclopedia of Dairy Sciences. 2002. Vol. 2.H.

7. Roginski H, Fuquay JW, Fox PF (Eds.). Encyclopedia of Dairy Sciences,v. 2, MPG Books Ltd, Bodmin, UK (2002), 630-637.

8. Egito AS, Girardet JM, Miclo L, Mollé D, Humbert G, Gaillard JL. Susceptibility of equine k - and b - caseins to hydrolysis by chymosin. Int Dairy J. 2001; 11:885–93.

9. Freitas MS, Durães MC, Freitas AF. Comparação da produção de leite e de gordura e da duração da lactação entre cinco "graus de sangue" originados de

cruzamentos entre Holandês e Gir em Minas Gerais. Arq. Bras. Med. Vet. Zootec. 2001; 53:708-713.

10. Galvão Júnior JGB, Rangel AHN, Medeiros HR., Silva JBA, Aguiar EM, Madruga RC, De lima júnior DM. Efeito da produção diária e da ordem de parto na composição físico-química do leite de vacas de raças zebuínas. Acta Vet. Brasílica. 2010; 4(1):25-30.

11. Gibbs PG, Potter GD, Blake RW, Mcmullan A. Milk production of Quarter horse during 150 days of lactation. J Anim Sci. 1982; 3(54):497-450.

12. Kramer JKG, Blackadar CB, Zhou J. Evaluation of two GC columns (60-m SUPELCOWAX 10 and 100-m CP sil 88) for analysis of milkfat with emphasis on CLA, 18∶ 1, 18∶ 2 and 18∶ 3 isomers, and short-and long-chain FA. Lipids. 2002; 37(8): 823-835.

13. Malacarne M, Martuzzi F, Summer A, Mariani P. Protein and fat composition of mare's milk: some nutritional remarks with reference to human and cow's milk. Int dairy J. 2002; 12:869-77.

14. Markiewicz-kęszycka, M.; Wójtowski, j.; kuczyńska, b.; puppel, k.; czyżak-runowska, g.; bagnicka, e.; krzyżewski, j. 2013. Chemical composition and whey protein fraction of late lactation mares' milk. Int dairy J. 2013; 31:62-64.

15. Markiewicz-kęszycka M, Wójtowski J, Czyżak-runowska G, Kuczyńska B, Puppel K, Krzyżewski J, Bagnicka E. Concentration of selected fatty acids, fat-soluble vitamins and β-carotene in late lactation mares' milk. Int dairy J. 2014; 38:31-36.

16. Nath LC, Anderson GA, Savage CJ, Mckinnon AO. Use of stored equine colostrum for the treatment of foals perceived to be at risk for failure of transfer of passive immunity. J Am Vet Med Assoc. 2010, 236:1085–1090.

17. National research council (NRC). Nutrient requirements of horses. 5. ed. Washington, D.C.: National Academy Press, 1989. 100p.

18. Orlandi M, Goracci J, Curadi MC. Fat composition of mare's milk with reference to human nutrition. Ann. Fac. Med. Vet. 2003, 56:97-105.

19. Pecka E, Dobrzański Z, Zachwieja A, Szulc T, Czyż K. Studies of composition and major protein level in milk and colostrum of mares. Anim. Sci. J. 2012, 83:162-168.

20. Pieszka M, Łuszczyński J, Szeptalin A. Comparison of mare's milk composition of different breeds. Nauka Przyroda Technologie, 2011 v. 5, v. 112.

21. Pieszka M, Łuszczyński J, Zamachowska M, Augustyn R, Długosz B, Hędrzak M. Is mare milk an appropriate food for people?–a review. Ann. Anim. Sci. 2016, 16:33-51.

22. Pikul J, Wójtowski J. Fat and cholesterol content and fatty acid composition of mares' colostrums and milk during five lactation months. Livest Sci. 2008, 113:285-290.

23. Pikul J, Wójtowski J, Danków R, Kuczynska B, Lojek J. 2008. Fat content and fatty acids profile of colostrum and milk of primitive Konik horses (Equus caballus gmelini Ant.) during six months of lactation. J Dairy Res, 2008, 75:302.

24. Pool-Anderson K, Raub RH, Warren JA. 1994. Maternal influences on growth and development of full-sibling foals. J Anim Sci. 1994, 72:1661-1666.

25. Reis ADP, Mesquita AJD, Moreira CHG, Curado EAF, Silva EBD, Nicolau ES. 2007. Composição do leite de éguas da raça Mangalarga Marchador. Rev. Inst. Adolfo Lutz, 2007, 66(2):130-135.

26. Salimei E, Varisco G, Rosi F. Major constituents, leptin, and non-protein nitrogen compounds in mares'colostrum and milk. Reprod Nutr Dev. 2002, 42:65–72.

27. Salimei S, Fantuz F. Equid milk for human consumption. Int Dairy J. 2012, 24:130-142.

28. Salamón RV, Salamón S, Csapó-kiss Z, Csapó J. Composition of mare's colostrum and milk I. Fat content, fatty-acid composition and vitamin contents. Acta Universitaria Sapientiae. 2009, 2(1):119-131.

29. Santos EM, Almeida FD, Vieira AA, Pinto LB, Corassa A, Pimentel RM, Silva VP, Galzerano L. Lactação em éguas da raça Mangalarga Marchador: produção e composição do leite e ganho de peso dos potros lactentes. R Bras Zootec 2005, 34(2):627-634.

30. Santos EM, Zanine AM. Lactação em éguas. Rev Port Ciênc Vet. 2006, 101:17-23.

31. Teodoro RL, Martinez ML, Verneque RS, Pires MFA. Parâmetros Genéticos e Fatores de Ajuste da Produção de Leite para o Efeito de Idade da Vaca ao Parto na Raça Guzerá. Rev. bras. Zootec. 2000, 29:2248-2252.
