# Supplementary material for: Chemical composition and lipid profile of mare colostrum and milk of the quarter horse breed
Source: PLoS One. 2020 Sep 14;15(9):e0238921. doi: 10.1371/journal.pone.0238921 (PMC7489553; doi:10.1371/journal.pone.0238921)
Supplement: Attachment — Submitted filename: revision letter PONE.docx [file pone.0238921.s002.docx]

Revision letter

PONE-D-20-04990 Chemical composition and lipid profile of mare colostrum and milk of the quarter horse

breed

Although the topic may be of interest,the manuscript lacks key elements:

The abstract contains unnecessary information; the keywords are not appropriate; the material and methods are not clear and lacking in information (eg. at 95 the authors do not explain how the groups were divided, they do not report the separation times of the foal for the colostrum; the amount of milk collected, at the time of samples, was not reported).

The statistical analysis should have consisted in ANOVA for repeated measurement and to include the effect of the farm. Furthermore, the two variables considered (age and parity (birth order) give similar information and one of the two variables should be removed from the model.

The references are not appropriated and are also dated (eg Cit 1, 2). In addition, eight tables are too numerous and the titles of the tables are not self-explanatory (eg Table 7).

Superfluous information is often reported in the text (e.g. l 106-110).

Finally, the results and conclusions are not satisfactory and should be reviewed in the light of the suggested considerations.
