# Supplementary material for: Chemical composition and lipid profile of mare colostrum and milk of the quarter horse breed
Source: PLoS One. 2020 Sep 14;15(9):e0238921. doi: 10.1371/journal.pone.0238921 (PMC7489553; doi:10.1371/journal.pone.0238921)
Supplement: Attachment — Submitted filename: Response to Reviewers 24.08.docx [file pone.0238921.s004.docx]

The authors thank the Reviewers and Editor for the considerations. The research group has been working hard to get the work published in the journal. The manuscript has been reviewed as suggested by editor and reviewers.

**Editor Comments:**

Needs to be checked native speaker English and all suggestions of reviewers should be addressed (even when you claim to have made changes). Thanks

The manuscript has been revided as suggested.

**Reviewer #1: (No Response)**

**Reviewer #2: Dear authors,**

I feel the paper could still benefit from further English editing and the revision has introduced sections that are difficult to read/comprehend. I have included some specific comments below, but am somewhat concerned that quite a few of my suggestions have not been addressed (even when you claim to have made changes). You have also referenced a number of papers that I cannot access and/or read. I assume these are relevant and have been cited appropriately.

The manuscript has been revided as suggested.

Specific comments/suggestions:

Title: Quarter Horse (captal Q and H)

The correction has been made.

43: just 'subjected' (not 'subjected instead of submitted')

The correction has been made.

45: the sentence has not been changed and I (still) think a comment should be added that the protein and lactose content are different to those (reported) in other breeds.

The correction has been made.

In the lines 42-43 the text has been modified as follow:

“There was a high protein content and reduced lactose content for the colostrum of the Quarter Horse mares, differing from other breeds”

71: just 'contains' (not 'is contains')

The correction has been made.

75: remove 'composed of'

The correction has been made.

78: is it 14% in milk and 29% in colostrum, please clarify

In the lines 79-80 the text has been modified as follow:

Regarding colostrum, which has a dry matter much higher than milk (14 in milk to 29% in colostrum)...

91: 'submitted for' (rather than 'submitted to')

The correction has been made.

92: please explain UFRN

The correction has been made.

104: dystocia instead of dystopian parts

The correction has been made.

105: were these removed from the group of 34 mares, or never included? please clarify

In the lines 110-112 the text has been modified as follow:

…In addition, mares, which gave birth to dead foals, were discarded from the experimental group as well, with 34 mares remaining in the experimental group at the end of the selection...

106: presumably collections took place within two seasons within this time frame. Please clarify

In the lines 112-114 the text has been modified as follow:

...The collections took place between the months of July/2017 and September/2018, because the 34 births were not concentrated in a single period...

180: 'in the colostrum' (rather than 'for the colostrum')

The correction has been made.

182: 'in Hungarian'

The correction has been made.

184: 'differed from that presented' (remove 'instead of those')

The correction has been made.

213: 'The fat content found in this study was lower...'

The correction has been made.

218: influenced or complicated rather than surrounded

The correction has been made.

222: 'nursing by the foal throughout'

The correction has been made.

242: I don't think it is correct to conclude that the fat% in your study is lower than the 0.9% reported by Burns, as both values will be a mean (with standard deviation) and you have not statistically compared the data. Maybe: seems to be slightly lower than the 0.9%.....

The correction has been made.

The sentence has been rewritten in line 263 as follow:

… of lactation, it seems to be slightly lower than the 0.9% reported by Burns et al. [17]...

267-270: I was hoping for a statement to indicate that not all changes found are likely to be biologically relevant (because they are so small, even if they are statistically relevant). It is not clear (to me) what you are trying to say in this sentence.

The correction has been made.

The sentence has been added in line 293 as follow:

...This can be an important detail in the management of herds that specialize in producing milk, or even in foal nutrition.

298: 'the present study' (instead of 'this study')

The correction has been made.

304: 'concentration' (instead of 'prevalence')

The correction has been made.

304: '...fatty acids in milk compared to colostrum'

The correction has been made.

307: 'range' (instead of 'threshold')

The correction has been made.

308: remove 'in fact'

The correction has been made.

314: 'results' (instead of 'result')

The correction has been made.

350: 'The milk composition is not influenced by...'

The correction has been made.

359: 'horses' instead of 'horse'

The correction has been made.

**Reviewer #3:** Authors did correct most of the proposed comments/changes.
However, I still consider (as two of my peers) that results should be separated from the discussion as explained before. When there are several peers that are addressing this poorly presentation of results, the author should consider to change it even it is not a mandatory requirement of PlosOne as answered by the authors.

This is why, I let the decision of accepting the article with a gathered results/discussion section to the editors.

The authors of this manuscript has been decided to keep the resultas and discussion together but it can modified as the Reviewer 2 suggested if it is a requirement of the Plos one journal.
